# Supplementary material for: Effects of Crop Resistance on the Tritrophic Interactions between Wheat Lines, Schizaphis graminum (Hemitera: Aphididae), and Propylaea japonica (Coleoptera: Coccinellidae)
Source: Plants (Basel). 2022 Oct 18;11(20):2754. doi: 10.3390/plants11202754 (PMC9611035; doi:10.3390/plants11202754)
Supplement: Supplementary file 1 [file plants-11-02754-s001.zip › plants-1892273-supplementary.pdf]

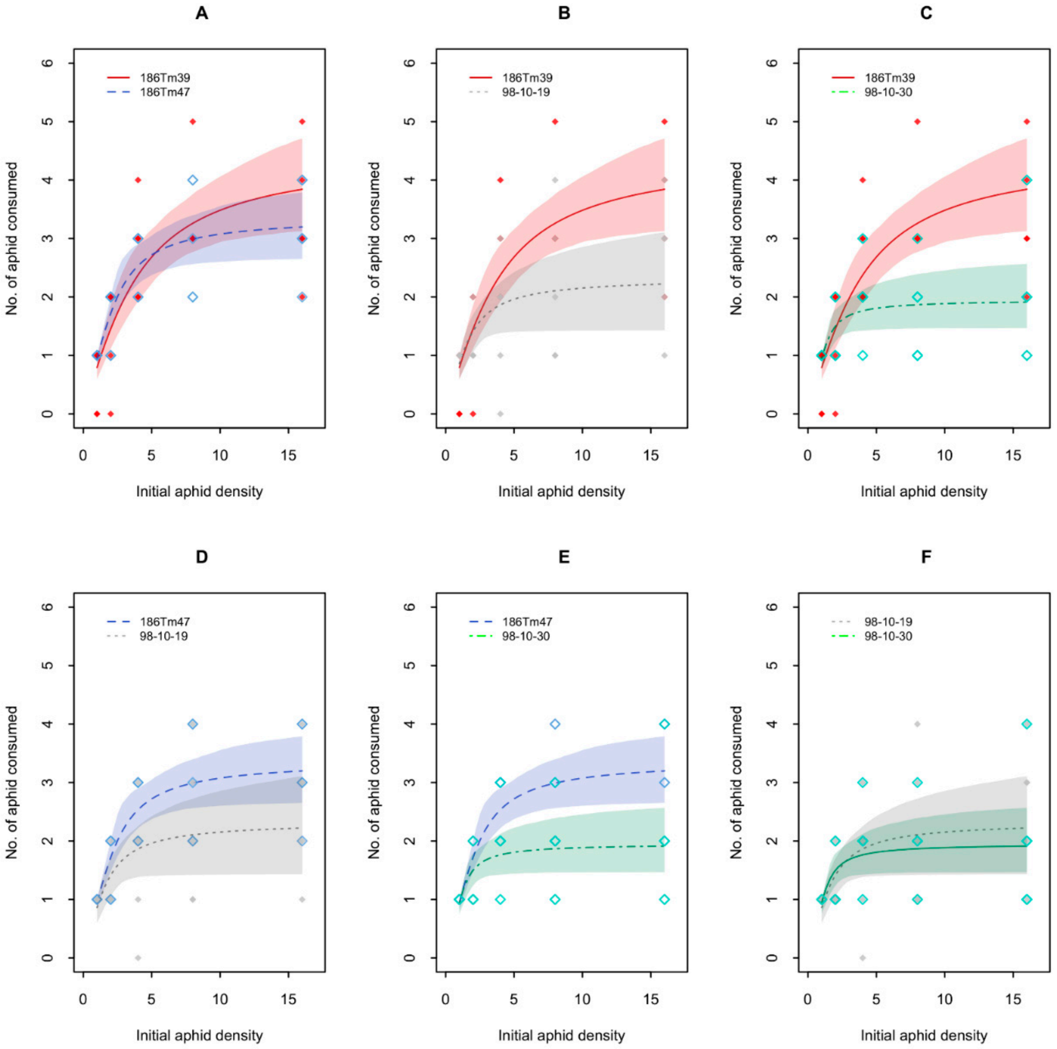

Figure S1: The functional responses curves of *Propylaea japonica* compared between prey from different wheat-acclimated *Schizaphis graminum* populations at first instar predators.

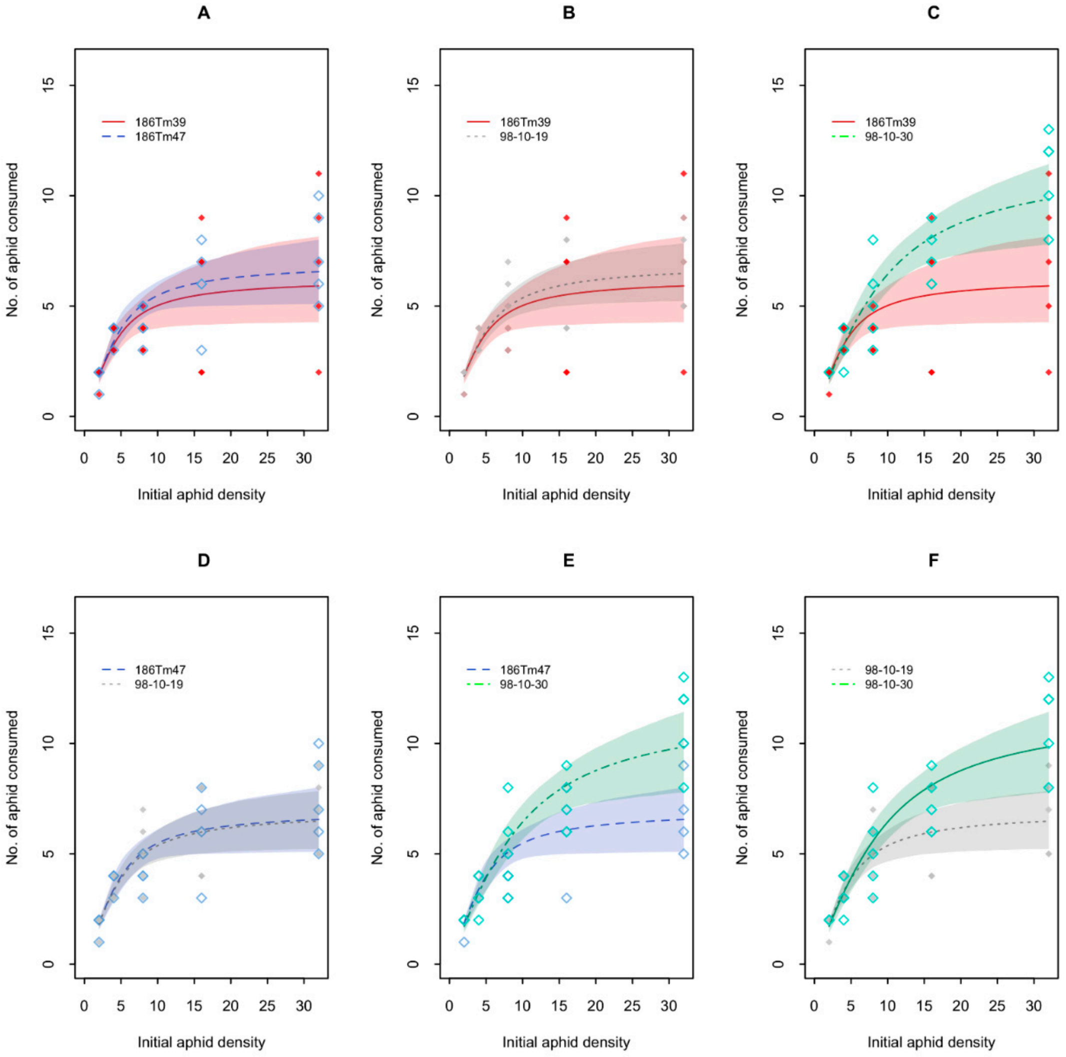

Figure S2: The functional responses curves of *Propylaea japonica* compared between prey from different wheat-acclimated *Schizaphis graminum* populations at second instar predators.

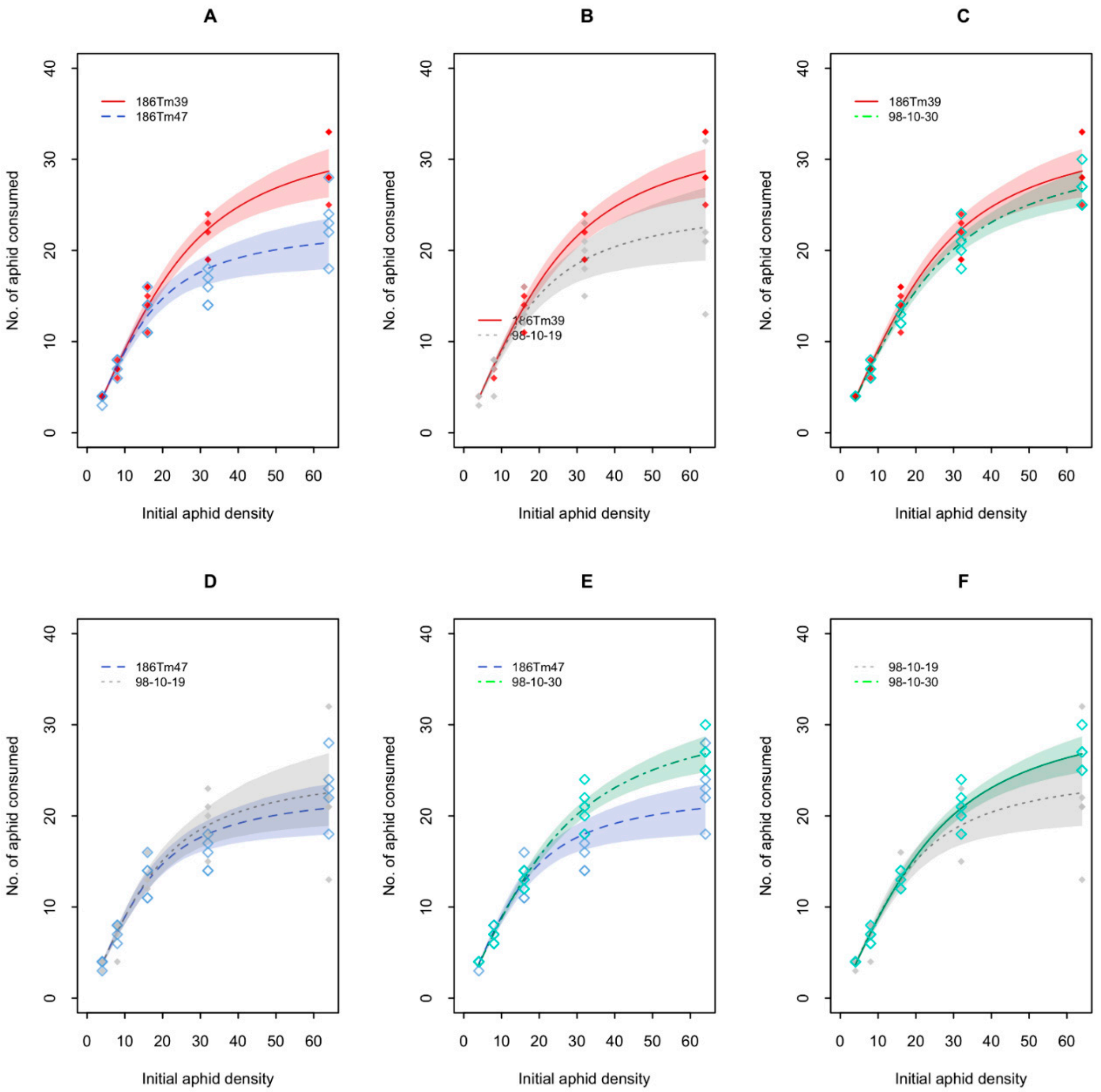

Figure S3: The functional responses curves of *Propylaea japonica* compared between prey from different wheat-acclimated *Schizaphis graminum* populations at third instar predators.

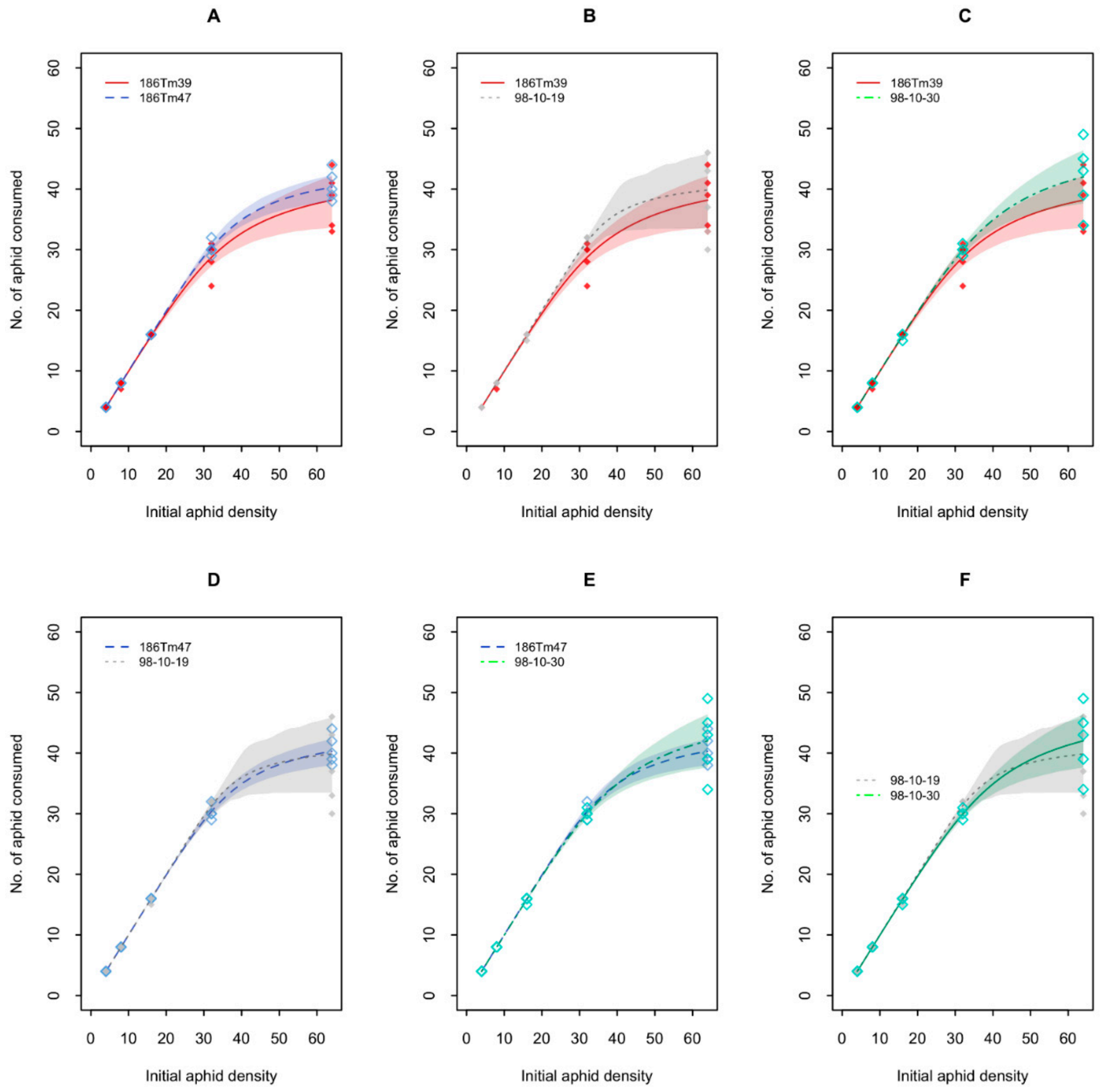

Figure S4: The functional responses curves of *Propylaea japonica* compared between prey from different wheat-acclimated *Schizaphis graminum* populations at fourth instar predators.

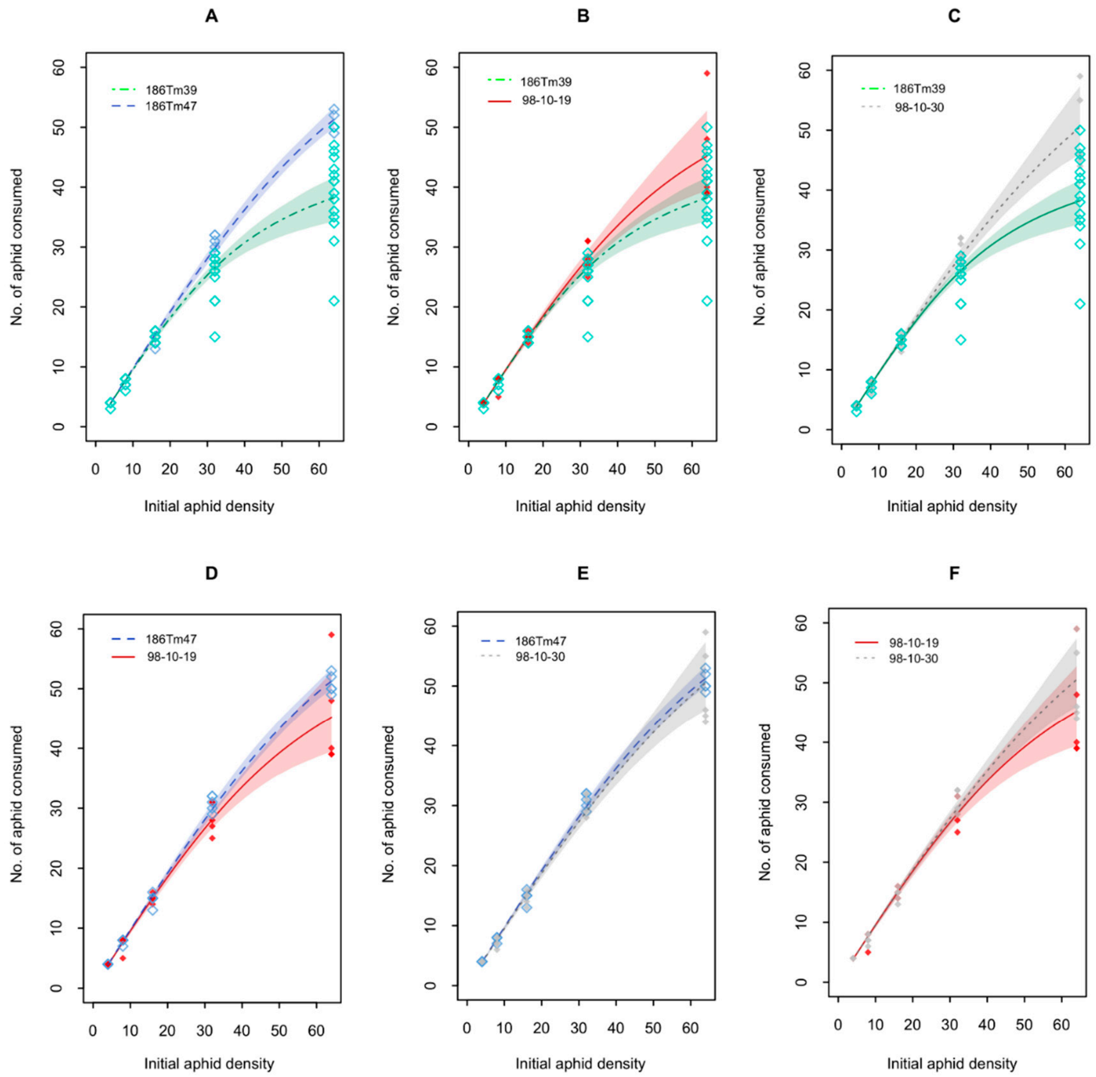

Figure S5: The functional responses curves of *Propylaea japonica* compared between prey from different wheat-acclimated *Schizaphis graminum* populations at female predators.

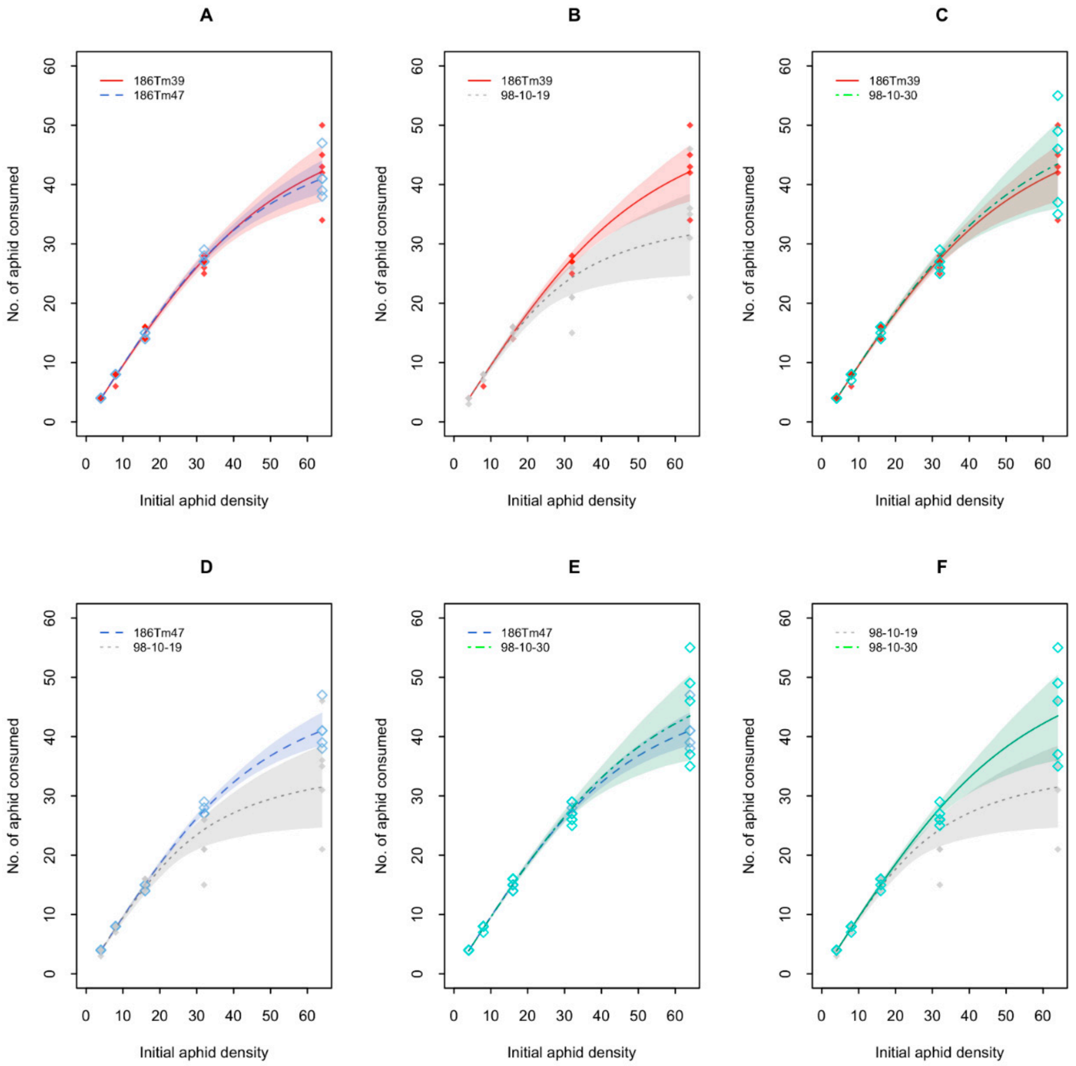

Figure S6: The functional responses curves of *Propylaea japonica* compared between prey from different wheat-acclimated *Schizaphis graminum* populations at male predators.

Table S1: The N_e and N_e/N_0 and their differences of each developmental stages predator feeding on 4 aphid acclimated population (Mean ± SE).

**Age stage**		**186Tm39**	**186Tm47**	**98-10-19**	**98-10-30**	***F***	***P***	**Populations pooled**
1st	N_e	2.32 ± 0.28 A	2.28 ± 0.21 A	1.72 ± 0.21 B	1.64 ± 0.16 B	5.12	0.004	1.98 ± 0.11 C
	N_e/N_0	52.75 ± 7.21	60.50 ± 6.47	48.75 ± 6.86	49.00 ± 6.80	1.30	0.28	49.79 ± 2.76 C
2nd	N_e	4.28 ± 0.53 B	4.64 ± 0.46 AB	4.52 ± 0.45 AB	5.72 ± 0.70 A	3.79	0.01	4.79 ± 0.27 C
	N_e/N_0	56.50 ± 6.55	59.63 ± 6.31	58.75 ± 6.25	64.88 ± 5.54	1.02	0.39	55.22 ± 2.45 C
3rd	N_e	15.24 ± 1.94 A	12.64 ± 1.42 B	13.08 ± 1.55 B	14.32 ± 1.77 AB	6.06	< 0.001	13.82 ± 0.83 B
	N_e/N_0	78.06 ± 4.22	71.06 ± 5.22	72.19 ± 5.15	74.75 ± 4.20	1.00	0.34	64.19 ± 1.97 B
4th	N_e	18.92 ± 2.65	19.76 ± 2.81	19.52 ± 2.76	19.96 ± 2.93	0.90	0.45	19.54 ± 1.37 A
	N_e/N_0	89.31 ± 3.29 b	91.56 ± 2.93 ab	91.56 ± 3.43 ab	91.63 ± 2.81 a	3.29	0.03	79.68 ± 1.54 A
Female	N_e	19.96 ± 3.17 b	21.64 ± 3.52 ab	21.20 ± 3.51 ab	22.28 ± 3.61 a	3.27	0.03	21.27 ± 1.70 A
	N_e/N_0	89.06 ± 2.81 B	93.12 ± 1.70 AB	90.81 ± 2.07 B	95.81 ± 1.55 A	4.71	0.004	78.78 ± 1.24 A
Male	N_e	19.24 ± 2.92 A	19.08 ± 2.80 A	16.52 ± 2.32 B	19.64 ± 3.06 A	4.44	0.007	18.62 ± 1.38 A
	N_e/N_0	88.00 ± 2.78 a	88.38 ± 2.74 a	81.94 ± 4.21 b	89.25 ± 2.69 a	2.70	0.05	74.42 ± 1.58 A
Age stage pooled	N_e	13.33 ± 1.07 AB	13.34 ± 1.10 AB	12.76 ± 1.05 B	13.92 ± 1.15 A	5.77	0.001	13.34 ± 0.55
	N_e/N_0	75.61 ± 2.31 AB	77.38 ± 2.19 A	74.00 ± 2.37 B	77.55 ± 2.20 A	4.47	0.004	67.01 ± 0.94

Note: Each parameter *d.f.* were 3, 80. The different capital letters ($P < 0.01$) and little letters ($0.01 < P < 0.05$) indicate the differences is significant among different acclimated populations based on Tukeys test in the left part of the table. The *d.f.* were 5, 80, and the different capital letters ($P < 0.01$) indicate the differences is significant among different age stages based on Tukeys test in the rightmost column of the table.

Table S2: Significance levels from linear-term logistic regression of the proportion of *Propylaea japonica* fed on four wheat-acclimated *Sch*
response parameters (*a* and T_h with mean, 95% CI)

Age stage	WAAP *	Linear coefficient	*Z* value	*P(Z)*	Response Type	Attack rate *a*	95% CI	*Z* value	*P*(Z)	Hand tim
1st	186Tm39	-0.154 ± 0.033	-4.66	<0.001	II	0.080±0.026	0.040-0.194	3.04	0.002	5.353±
	186Tm47	-0.177 ± 0.035	-5.09	<0.001	II	0.166±0.079	0.081-0.763	2.10	0.036	7.085±
	98-10-19	-0.140 ± 0.035	-4.01	<0.001	II	0.130±0.100	0.043-0.798	1.31	0.191	10.287
	98-10-30	-0.183 ± 0.038	-4.80	<0.001	II	0.247±0.244	0.076-2.311	1.01	0.310	12.277
2nd	186Tm39	-0.073 ± 0.012	-6.78	<0.001	II	0.145±0.054	0.061-0.604	2.67	0.008	3.822±
	186Tm47	-0.075 ± 0.012	-6.41	<0.001	II	0.154±0.054	0.072-0.491	2.82	0.005	3.434±
	98-10-19	-0.082 ± 0.012	-6.79	<0.001	II	0.142±0.044	0.081-0.270	3.19	0.001	3.457±
	98-10-30	-0.060 ± 0.011	-5.38	<0.001	II	0.096±0.025	0.055-0.179	3.87	<0.001	2.047±
3rd	186Tm39	-0.039 ± 0.004	-8.69	<0.001	II	0.138±0.020	0.100-0.195	6.88	<0.001	0.686±
	186Tm47	-0.038 ± 0.004	-9.10	<0.001	II	0.149±0.027	0.101-0.228	5.55	<0.001	1.024±
	98-10-19	-0.044 ± 0.004	-10.02	<0.001	II	0.141±0.022	0.087-0.222	6.43	<0.001	0.928±
	98-10-30	-0.037 ± 0.004	-8.63	<0.001	II	0.118±0.017	0.097-0.150	6.99	<0.001	0.724±
4th	186Tm39	-0.038 ± 0.006	-8.53	<0.001	II	0.270±0.046	0.170-0.454	5.88	<0.001	0.541±
	186Tm47	-0.080 ± 0.010	-7.68	<0.001	II	0.376±0.079	0.299-0.569	4.74	<0.001	0.530±
	98-10-19	-0.137 ± 0.025	-5.45	<0.001	II	0.606±0.137	0.259-8.603	4.42	<0.001	0.562±

	98-10-30	-0.070 ± 0.009	-7.60	<0.001	II	0.289±0.048	0.216-0.416	6.00	<0.001	0.483±0.034	0.401-0.586	14.27	<0.001
female	186Tm39	-0.038 ± 0.006	-6.23	<0.001	II	0.149±0.018	0.101-0.203	8.39	<0.001	0.349±0.037	0.086-0.459	9.32	<0.001
	186Tm47	-0.041 ± 0.008	-5.26	<0.001	II	0.173±0.021	0.129-0.234	8.13	<0.001	0.286±0.033	0.210-0.339	8.58	<0.001
	98-10-19	-0.032 ± 0.007	-4.87	<0.001	II	0.143±0.016	0.110-0.204	8.68	<0.001	0.259±0.037	0.095-0.363	7.09	<0.001
	98-10-30	-0.040 ± 0.003	-12.94	<0.001	II	0.155±0.012	0.127-0.188	12.95	<0.001	0.475±0.026	0.397-0.577	18.27	<0.001
male	186Tm39	-0.038 ± 0.006	-6.67	<0.001	II	0.149±0.019	0.113-0.201	7.96	<0.001	0.398±0.041	0.277-0.515	9.74	<0.001
	186Tm47	-0.043 ± 0.006	-7.32	<0.001	II	0.165±0.021	0.141-0.195	7.79	<0.001	0.434±0.040	0.342-0.484	10.94	<0.001
	98-10-19	-0.040 ± 0.005	-8.28	<0.001	II	0.166±0.027	0.102-0.269	6.23	<0.001	0.633±0.057	0.413-0.881	11.12	<0.001
	98-10-30	-0.037 ± 0.006	-6.35	<0.001	II	0.154±0.020	0.115-0.212	7.76	<0.001	0.382±0.040	0.220-0.538	9.43	<0.001

Note: *, WAAP is wheat line-acclimiated aphid populaiton.
